# Synthetic scaffolds for musculoskeletal tissue engineering: cellular responses to fiber parameters

**DOI:** 10.1038/s41536-019-0076-5

**Published:** 2019-06-27

**Authors:** Thomas Lee Jenkins, Dianne Little

**Affiliations:** 1 Department of Biomedical Engineering, Purdue University, West Lafayette, IN 47907 USA; 2 Department of Basic Medical Sciences, Purdue University, West Lafayette, IN 47907 USA

**Keywords:** Tissues, Tissue engineering, Biomaterials - cells

## Abstract

Tissue engineering often uses synthetic scaffolds to direct cell responses during engineered tissue development. Since cells reside within specific niches of the extracellular matrix, it is important to understand how the matrix guides cell response and then incorporate this knowledge into scaffold design. The goal of this review is to review elements of cell–matrix interactions that are critical to informing and evaluating cellular response on synthetic scaffolds. Therefore, this review examines fibrous proteins of the extracellular matrix and their effects on cell behavior, followed by a discussion of the cellular responses elicited by fiber diameter, alignment, and scaffold porosity of two dimensional (2D) and three dimensional (3D) synthetic scaffolds. Variations in fiber diameter, alignment, and scaffold porosity guide stem cells toward different lineages. Cells generally exhibit rounded morphology on nanofibers, randomly oriented fibers, and low-porosity scaffolds. Conversely, cells exhibit elongated, spindle-shaped morphology on microfibers, aligned fibers, and high-porosity scaffolds. Cells migrate with higher velocities on nanofibers, aligned fibers, and high-porosity scaffolds but migrate greater distances on microfibers, aligned fibers, and highly porous scaffolds. Incorporating relevant biomimetic factors into synthetic scaffolds destined for specific tissue application could take advantage of and further enhance these responses.

## Introduction

Tissue engineering uses engineering and life science structure–function relationships to restore, preserve, or improve tissue function. Understanding the interactions between cells and their extracellular matrix (ECM) is critical for this process. The ECM provides structural support to the cells and provides cues for regulating cell differentiation, attachment and morphology, migration, and immune response. The major components include proteoglycans and fibrous proteins. Proteoglycans regulate and maintain the ECM. For example, in cartilage and tendon, decorin^1^ and biglycan^2^ regulate collagen fibrillogenesis. In tumors, syndecans influence growth and invasion, and perlecan promotes angiogenesis.^3^ While in neurons, heparan-sulfate proteoglycans enhance neurite outgrowth, but chondroitin-sulfate proteoglycans inhibit neurite outgrowth.^4^ While proteoglycans have many vital functions, some of which remain undefined, fibrous proteins comprise the most abundant portion of the ECM. This review highlights the characteristics of fibrous ECM proteins and of fabrication methods for fibers and model systems used in musculoskeletal tissue engineering, with comparison to other tissues and cell-based systems where gaps in the literature were identified. Finally, this review examines the relationship between the fiber parameters of tissue engineered scaffolds and the cell responses (i.e., differentiation, morphology, and migration) elicited.

## Major fibrous proteins in the extracellular matrix

**Fig. 1:**
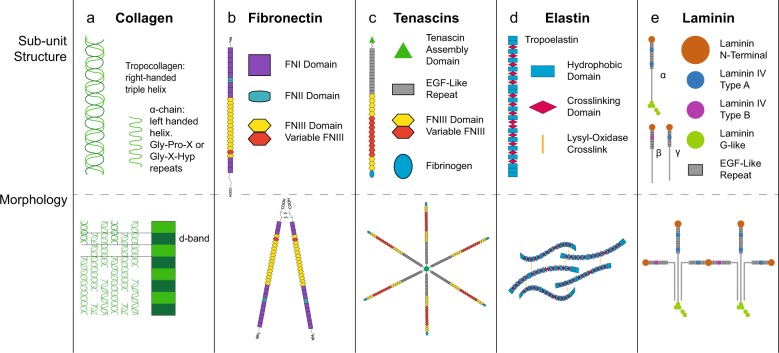
Fibrous proteins of the extracellular matrix. **a** The basic unit of collagen fibrils is the tropocollagen triple-helix comprised of three α-chains. Each α-chain forms a helix where glycine is positioned at every third amino acid, often with glycine-proline-X or glycine-X-hydroxyproline repeats. Tropocollagen molecules form collagen fibrils by binding together in a quarter-stagger pattern that gives collagen its characteristic banding pattern. Collagen fibrils vary in diameter, alignment, and packing depending on the tissue they are found in. **b** Fibronectin (FN) polypeptide chains are comprised of three variable domains: FNI, FNII, and FNIII. Each polypeptide chain contains 12 FNI domains, 2 FNII domains, and 15–17 FNIII domains. Pre-mRNA splicing produces at least 20 variants of the protein in humans. Fibronectin polypeptide chains form a ‘V’ shape at the C-terminus via two disulfide bonds. Fibronectin is secreted as a globular protein that is stretched by cells into its fibrillar form. **c** Tenascin fibrils are comprised of varying numbers of heptad repeats, epidermal growth factor (EGF)-like repeats, fibronectin type III (FNIII) domains, and a globular fibrinogen domain capping the C-terminus. Tenascin fibrils bind at the N-terminus to form hexamers and trimers. **d** Tropoelastin molecules contain alternating hydrophobic domains and crosslinking domains. Elastin fibers are generally relaxed and coiled. Lysyl-oxidase crosslinks the fibers together to form a network. When the tissue is stressed, the elastin uncoils and elongates. **e** Each laminin contains an α-chain, a β-chain, and a γ-chain. There are five α-chain, four β-chain, and three γ-chain variants. Each chain contains a combination of laminin N-terminal domains, laminin IV type A domains, laminin IV type B domains, and EGF-like repeats. α-chains contain laminin G-like domains at the C-terminal of the peptide. Laminins form helical glycoproteins composed of three polypeptide chains (α, β, γ). There are 15 known combinations of α-, β-, and γ- chains. Three short chains (α, β, γ) at the N-terminal interact with the ECM, and a long chain (α) at the C-terminal binds to cell-membrane integrins. Laminin 111 shown

Collagen is the most abundant protein in the body^5^ and while 28 types of collagen have been discovered to date,^6^ not all collagens are fibril-forming. The fibrillar collagens include types I, II, III, V, XI, XXIV, and XXVII. Type I collagen is the most abundant of all,^7^ comprising significant portions of the ECM in bone,^8^ tendon,^6^ ligament,^9^ skin,^10^ and blood vessels,^11^ where fibril alignment begets function. In tendon ECM, collagen molecules form a hierarchal structure of aligned, tightly packed fibrils (50–500 nm diameter), fibers (1–20 μm diameter), and fascicles (50–300 μm diameter).^12^ In contrast, type II collagen fibers in articular cartilage form differentially aligned networks in each of three zones: superficial, intermediate, and deep. In the superficial zone, type II collagen fibers align parallel to the surface and pack densely. In the intermediate zone, the collagen fibers are thicker and randomly oriented. In the deep zone, the largest of the collagen fibers align perpendicular to the surface. Type I collagen fibers also support the myofibrils in muscle^13^ and are a major component of bone and blood vessels, forming a concentric weave pattern.^14^ Despite the abundance of collagen in the body, substantial gaps remain in understanding its interactions with cells.^15^ (Fig. 1 and Table 1)

**Table 1:** Fibrous proteins of the extracellular matrix integrin binding, cellular interactions, and knockout and mutation effects

	Collagen	Fibronectin	Tenascins	Elastin	Laminin
Integrin Binding Partners	α_1_β_1_	α_5_β_1_	α_9_β_1_ (TNC)	α_V_β_3_	α_3_β_1_
	α_2_β_1_	α_v_β_3_	α_11_β_1_ (TNX)	α_V_β_5_	α_6_β_1_
	α_10_β_1_		α_4_β_1_ (TNR)		α_7_β_1_
	α_11_β_1_		α_5_β_1_ (TNR)		α_6_β_4_
Cellular Interactions	Adhesion	Adhesion to ECM	Adhesion Modulator	Adhesion	Adhesion
	Differentiation	Migration	− anti-FN adhesion (TNC)	Proliferation	Migration
	Wound Healing	Wound Healing	− pro-neurite adhesion (TNR)	Chemotaxis	
	ECM Organization	Growth	Proliferation (TNC)		
		Differentiation	Migration (TNC)		
Knockout effects	Lethal (I)	Lethal (FN1)	CNS Abnormalities (TNC, TNR)	Lethal	Lethal
	Ehlers-Danlos (III)		Behavioral Difference (TNR)		
			Ehlers-Danlos (TNX)		
Mutation effects	Ehlers-Danlos (I, III, V)	Glomerulopathy	CNS Abnormalities (TNC, TNR)	Supravascular aortic stenosis	Poretti-Boltshauser
	Osteogenesis Imperfecta (I)		Behavioral Difference (TNR)	Williams-Beuren Syndrome	Cerebellar Dysplasia
	Chondrodysplasia (II)		Ehlers-Danlos (TNX)	Cutis Laxa	
	Atopic Dermatitis (III)				
	Caffey Disease (I)				

*CNS* Central Nervous System *I* type I collagen, *II* type II collagen, *III* type III collagen, *V* type V collagen, *FN1* fibronectin domain 1, *TNC* tenascin C, *TNR* tenascin R, *TNX* tenascin X

Fibronectin is a glycoprotein that connects cells to the ECM.^16^ Fibronectin exists in two conformations: globular and fibrillar.^17^ Following secretion, α_5_β_1_ and α_5_β_3_ integrins stretch fibronectin into the fibrillar form. Fibronectin domains form ligand binding sites to proteins such as collagens, proteoglycans, fibrins,^16^ and multiple integrins.^18^ Beyond adhesion to the matrix, fibronectin provides a means for cells to assemble^19^ and regulate the ECM. Fibronectin affects cell migration,^20^ which has implications for wound healing^21^ and disease.^22^

Tenascins are a family of fibrillar glycoproteins (-C, -R, -W, -X).^23^ Tenascin-C is found mostly in musculoskeletal tissues including the myotendinous junction^24^ and is expressed during development and wound healing.^24^ Tenascin-R is expressed solely in the central nervous system.^25^ Tenascin-X is expressed in muscle and skin.^26^ Tenascin-W is present in kidney and smooth muscle^26^ and is a biomarker of solid tumors.^25^

Elastin is a fibrous protein that maintains tissue elasticity, and therefore, is crucial in arteries, the lungs, skin, tendon, and ligaments.^27^ Elastin forms when tropoelastin, a precursor protein secreted by cells, has its signal peptide cleaved and polymerizes.^28^ Lysyl-oxidase cross-links allow the elastin network to stretch and relax without deformation.^29^ Elastin regulates cell proliferation, promotes adhesion, and is a chemotactic agent.^30^

Laminins are vital to the basal membrane, which surrounds neural tissue, endothelium and epithelium, muscle cells, and fat cells, among other tissues.^31^ Fifteen laminin isoforms have been discovered in humans, with genes for five α-chains, three β-chains, and three γ-chains identified.^32^ Laminins regulate cell adhesion and migration, transmitting forces from the ECM through integrins and focal adhesions to the actin cytoskeleton in a manner distinct from collagen and fibronectin: laminin-integrin binding leads to smaller and fewer focal adhesions and actin stress fibers, which enhances cell migration.^33^

In summary, fibrous proteins provide many binding motifs for cell adhesion and a supportive framework for cell growth. They transmit forces from the ECM through the cell to regulate gene expression, cell migration, and cell spreading. Tissue engineering, therefore, seeks to develop and refine biomaterials that mimic the fibrous ECM to enhance intended cellular responses using an understanding of mechanisms of cell-fiber interactions gained from using model fiber systems.

## Tissue engineered scaffolds

Tissue engineered scaffolds provide a structural framework that resembles the fibrous protein component of the ECM. There are several approaches to scaffold fabrication: natural polymers produced by cells, synthetic polymers, or a combination thereof. Natural polymers provide relevant biomimetic properties and cell signaling cues but offer little control over the scaffold structural or architectural properties, i.e., fiber diameter, alignment, or porosity. Conversely, synthetic polymers provide improved control over the scaffold structure and micro-architecture, but few matrikines or other biomimetic cues, without additional process engineering. Finally, both three-dimensional (3D) scaffold systems and more simple one (1D) and two (2D) dimensional models can examine mechanisms of cell interactions with fibers to inform larger scale fabrication methods.

**Fig. 2:**
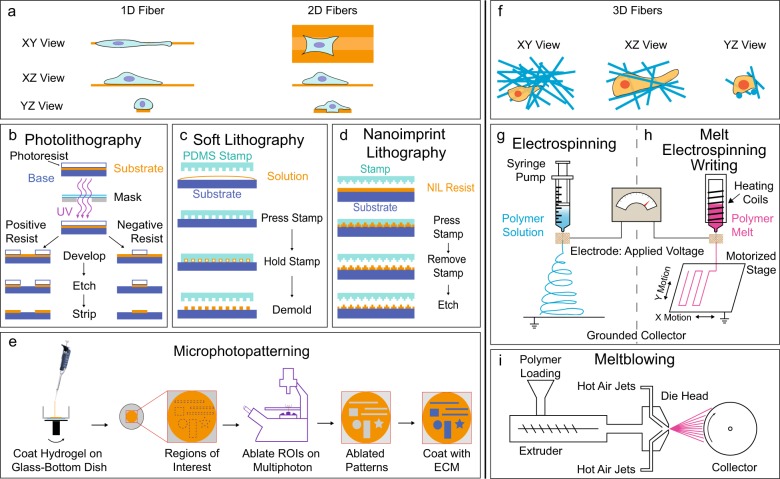
Methods for preparing synthetic polymer scaffolds. 1D/2D Scaffolds **a**. In photolithography **b** a substrate is covered with a light-sensitive organic material termed a positive or negative photoresist. The photoresist is then exposed to a specific pattern of intense UV radiation. With positive photoresist, UV light causes the exposed photoresist to become soluble, allowing removal with solutions known as developers. For a negative photoresist, UV light causes the exposed regions to become insoluble, and the shielded photoresist is removed with developers. The remaining photoresist is removed by etching to create the desired scaffold. In soft lithography **c** a pattern master and a stamp pattern the substrate. Masters are commonly produced using photolithography, or through electron beam lithography if greater resolution is desired. The masters are used to form the stamps, often using an elastomeric polymer. The stamp is then pressed into a solution to create the desired pattern on the substrate. In nanoimprint lithography **d** a silicon stamp (of the inverse pattern) is used to imprint a pattern into the desired substrate at a specific temperature and pressure. The mold is then removed leaving the model fiber system on the desired substrate. In microphotopatterning **e** a thin hydrogel is spin-coated onto a prepared glass-bottom dish. The hydrogel is ablated using a multiphoton microscope in prescribed regions of interest created using microscope-specific imaging software. Next, the ablated regions are functionalized using ECM proteins, commonly fibronectin, to allow cell adhesion to the patterns. 3D Scaffolds **f**. In electrospinning **g** a positively charged polymer solution is extruded through an orifice, where it forms a jet. The jet elongates toward a grounded collector, the solvent evaporates, and polymer fibers are drawn out towards the collector. In melt electrospinning writing **h**, the polymer is melted rather than in solution. The melted polymer is extruded through a high-voltage electric field toward a grounded, motorized stage to collect the scaffold. In meltblowing **i** a melted polymer is extruded through die heads known as spinnerets, where hot air jets attenuate the polymer melt into fibers that form a sheet of fabric as they cool and crystalize

Lithography involves printing a pattern into a flat synthetic polymer surface using one of several variations to the basic method (see Fig. 2b–d for some common methods of lithography). Lithography methods offer consistent, easy to produce 1D and 2D systems, with highly controllable fiber parameters (Table 2). However, changing the pattern master is nontrivial and time-consuming.

**Table 2:** Fiber parameters of synthetic scaffolds

	2D Scaffolds				3D Scaffolds		
	PL	SL	NIL	μPP	ES	MES	MB
Diameter							
Min	300nm	30nm	10nm	<1μm	5nm	270nm	500nm
Max		100μm		xFOV	10μm	500μm	>30μm
Alignment	0–180°	0–180°	0–180°	0–180°		0–180°	
Porosity	0–100%	0–100%	0–100%	0–100%	0–90%	0–100%	70–90%

*PL* photolithography, *SL* soft lithography, *NIL* nanoimprint lithography, *μPP* microphotopatterning, *ES* electrospinning, *MES* melt-electrospinning, *MB* meltblowing, *xFOV* multiples of field of view

Microphotopatterning produces 1D and 2D fiber systems using a multiphoton microscope^34^ (Fig. 2e). Using computer-software generated patterns makes it easy to alter the master pattern. The fiber parameters are limited by the microscope’s resolution (Table 2). Microphotopatterning provides a simple method to study cellular mechanisms of migration^35^ and matrix production^36^ and the use of glass-coverslip bottomed dishes facilitates various imaging, histological or immunostaining techniques.^34^

Electrospinning produces 3D scaffolds by using an electric field (Fig. 2g). Randomly oriented fiber scaffolds are collected on a grounded solid or liquid media.^37^ Aligned fiber scaffolds are formed in a variety of ways: using a rotating disc^38^ or mandrel,^39^ patterned electrodes,^40^ air-gap techniques,^41^ a patterned insulator,^42^ or high strength ceramic or electromagnets with copper plates.^43^ Electrospinning is used to produce prototypical biomaterial scaffolds for tissue engineering for tendon,^44,45^ bone,^46^ cartilage,^47,48^ meniscus,^49^ smooth and skeletal^50^ muscle, and neural tissue^51,52^ and produces highly tunable scaffold structure and architecture. Electrospinning parameters such as voltage, flow rate, distance to the collector, polymer concentration, solution conductivity, solvent, humidity, and temperature determine the fiber characteristics.^53^ Fibers parameters can be finely tuned (Table 2). The addition of a second electrode and a 2D moving platform allows for focused fiber control and custom-defined, open-pore scaffold architectures, which is a promising technique for creating tissue microenvironments.^54^ Electrospun scaffolds are also fabricated using recombinant protein-based polymers, such as elastin-like recombinamers,^55^ which are biocompatible and allow for incorporating protein functional domains (e.g., arginine-glycine-aspartic acid (RGD) peptide motif) into the fibers. However, while electrospinning offers substantial control over the scaffold parameters, it produces small volumes of scaffold at low rates.^53,56^ Furthermore, electrospinning requires the use of solvents,^53^ which can be toxic. Nonetheless, electrospinning is a versatile method to produce fibers for tissue engineering, as reviewed recently.^57^

Melt electrospinning is similar to electrospinning, but the polymer is melted rather than in solution (Fig. 2h). However, after the melted polymer enters the electric field, due to the melt’s high viscosity and the proximity to the collector, the polymer jet moves to the collector in a more controlled manner than in electrospinning as it cools and crystallizes. Therefore, melt-electrospinning creates highly structured scaffolds. Furthermore, adding moving collectors defines a technique known as melt electrospinning writing (MEW), a form of 3D printing (Fig. 2h). Melt electrospinning offers the advantages of predictable scaffold structure and architecture, along with the use of no solvents (see Table 2 for fiber parameters). As for electrospinning, in melt electrospinning, the flow rate, the extrusion needle diameter, and molecular weight control fiber diameter, but in MEW, the pressure in the air gap and the collector speed collector provides additional control.^58^ In all forms of electrospinning, distance to the collector and voltage at the extrusion needle or nozzle influence fiber properties, but the effect of voltage on the fiber properties is debatable for melt electrospinning.^59^ Melt electrospinning and MEW have been used to produce scaffolds for bone,^58^ cartilage,^60^ cardiovascular,^61^ dermal,^62^ and neural^63^ tissue engineering.

Wet spinning is one of the oldest methods of producing fibers from polymers but is less commonly used for tissue engineering. In wet spinning, the polymer is dissolved using a non-volatile solvent. The solvent is then drawn out by a chemical reaction or washed out through spinnerets into a bath. The solvent is then removed in a liquid coagulation medium, leaving the polymer, which forms fibers. Wet spinning has mostly been used as a scaffold for bone tissue engineering,^64^ but it has also been used for soft tissue,^65^ vascular,^66^ dermal,^67^ and neural tissue engineering,^68^ as well as for wound dressings^69^ and drug delivery.^70^ While wet spinning is a more rapid fabrication process (7–150 m/min) than electrospinning, it requires multiple wash steps to remove processing impurities.

Meltblowing has recently been re-purposed to produce synthetic polymer scaffolds in tissue engineering. First patented in the 1930s,^71^ meltblowing (Fig. 2i) is used to produce materials like surgical drapes, hygiene products, and filtration devices.^72^ In recent work, meltblowing has been used in neural,^73^ vascular,^61^ bone,^74^ adipose,^74^ and tendon tissue engineering.^75^ Meltblowing has a major advantage over electrospinning as it can produce fabrics at rates up to 5000 m/min^56^ and requires no harsh solvents. Fabrics produced by meltblowing have fibers diameters from 500 nm^76^ to hundreds of microns^77^ in diameter (Table 2), exhibit alignment and anisotropy,^75^ and range in porosity from 70 to 99%.^78^ Meltblowing is a promising method for studying cellular mechanisms and tissue response, with proven high-throughput scalability for translation.

## General fiber parameters

While multiple processes are used to fabricate synthetic polymer scaffolds to support cells in tissue engineering, there is a need to understand how various scaffold parameters such as fiber diameter, alignment, and porosity guide cell response, proliferation, and differentiation. Fiber diameters found in synthetic scaffolds can range from nanofibers (<1 μm) to microfibers (≥1 μm). Fiber alignment can range from randomly oriented fibers, intersecting in all directions, to tightly aligned fibers running in parallel; the ability to tune alignment within narrow parameters varies with the fabrication method used. Porosity is the amount of space in the volume of a material, which can theoretically range from 0 to 100% in 3D fibrous scaffolds. In 1D and 2D model systems, the ‘fibers’ are often channels: an anti- or ‘inverted’ fiber. Therefore, the ‘fiber diameter’ of a 1D/2D model system represents the channel width. The alignment of 2D model systems describes the angles between the channels. The porosity describes the spacing between the channels.

Finally, when examining the effects of fiber parameters on cell differentiation and gene expression, it is important to consider whether the culture media is a differentiation or an induction media and if serum is used. Differentiation media contains growth factors and other biomolecules to promote the differentiation of cells into specific cell types. Induction media also includes factors to induce specific responses of cells, which can include differentiation, but can also influence other responses, including cell behavior, gene expression, morphology, or signaling cascades. The types of media must be compared within and between studies to determine whether the observed effects are caused by the media or by the fiber parameters.

## General fiber parameters: Fiber diameter

Fiber diameter undoubtedly modulates cell differentiation. Human, adipose-derived mesenchymal stem cells (hASCs), without specific differentiation or induction media, underwent osteogenesis on 2 μm microfibers but adipogenesis on 15 μm microfibers.^79^ However, hASCs exhibited no upregulation of markers of osteogenesis, adipogenesis, chondrogenesis, or myogenesis on nanofibers (650 nm diameter) without differentiation media.^79^ In contrast, human bone marrow-derived mesenchymal stem cells (hMSCs) increased expression of osteogenic markers *RUNX2*,^80^ osteocalcin (*OCN*),^80,81^ alkaline phosphatase (*ALP*),^81^ and osteopontin (*OPN*)^81^ on nanofibers (<400 nm) compared to microfibers (1.4–4 μm).

Fiber diameter influences chondrogenesis. hMSCs exhibited dual differentiation potential on microfibers (3–14 μm in diameter), expressing both osteogenic marker *RUNX2* and chondrogenic marker SRY-box 9 (*SOX9)* without induction media but expressed osteogenic or chondrogenic markers with their respective differentiation media.^48^ hMSCs in chondrogenic differentiation media increased expression of chondrogenic markers aggrecan (*ACAN*) and type II collagen and a higher type II:I collagen ratio on microfibers ~4 μm diameter compared to hMSCs on nanofibers.^47^ However, bovine chondrocytes increased expression of chondrogenic markers *SOX9* and *SOX5* and a higher type II:I collagen ratio on nanofibers (400 nm diameter) compared to larger nanofibers (700 nm) and thin microfibers (1.33 μm).^82^ In a 2D nanoimprint lithography fiber system, hMSCs exhibited chondrogenesis on smaller nanofiber (250 nm) channels.^83^ Therefore, both smaller nanofibers (<400 nm) and larger microfibers (3–14 μm) can stimulate greater chondrogenesis than intermediate fiber diameters.

Fiber diameter has a time-dependent relationship with tenogenesis. In a 2D photolithography system, at day 1, human tenocytes upregulated tendon-related genes (type I and II collagens, scleraxis, tenomodulin, and tenascin-C) on 2 μm fibers compared to 37 nm or 317 nm fibers.^84^ However, at day 5 gene expression flipped: nanofibers upregulated tendon-related genes compared to the microfibers.^84^ Similarly, in electrospun scaffolds, tendon-related genes type I collagen and decorin were upregulated on nanofibers (280 nm diameter) compared to larger fibers (820 nm and 2.3 μm) at day 7, with no difference in tenomodulin or scleraxis expression.^85^ As time progressed, types I, III and V collagen, along with tenomodulin, were upregulated on microfibers with a 1.8 μm diameter compared to nanofibers, even though there was more collagen in the matrix on the nanofibers compared to the microfibers,^86^ highlighting the importance of considering both protein and gene expression together. Tenogenesis appears to favor microfibers initially,^84^ followed by nanofibers beginning at around one week,^84,85^ before favoring microfibers again at later time points.^86^ The reasons for this are unknown but could represent pre-programmed attempts to repair injuries of different severity or cellular responses to different fibrillar proteins.

Fiber diameter also modulates stem cell differentiation into different neural cell types. Rat neural stem cells (rNSCs) differentiated into glial cells (increased oligodendrocyte marker RIP-antigen) on 238 nm fibers. However, rNSCs differentiated into neuronal cells (increased neuronal marker β-III tubulin (Tuj-1) expression) on 1.452 μm fibers in neuronal differentiation medium.^87^ Human embryonic stem cell-derived neural precursors experienced more robust differentiation into neuronal cells on 400 nm fibers than on 800 nm fibers in the presence of neuronal differentiation media.^52^ While there appears to be a discrepancy as to how fiber diameter guides neural differentiation, these studies used different cell types from different species and different differentiation media, complicating comparison. Additionally, the cellular response to fiber diameter could depend on differentiation state.

Greater differentiation of human embryonic stem cells (hESCs) increased expression of definitive endoderm genes goosecoid homeobox (*GSC*), mix paired-like homeobox 1 (*MIXL1*), C-X-C motif chemokine receptor 4 (*CXCR4*), *SOX17*, and forkhead box A2 (*FOXA2)* with downregulation of primitive endoderm marker *SOX7* on 200 nm fibers compared to 500 nm, 800 nm, or 1.3 μm fibers.^88^ This upregulation of definitive endoderm markers persisted on 200 nm fibers both as single cells and as cell clusters.^88^

Fiber diameter also maintains stemness. hMSCs increased gene expression of pluripotency markers nanog homeobox (*NANOG*) and octamer-binding transcription factor 4 isomer A (*OCT4A)* on 290 nm nanofiber 2D system, suggesting that nanofibers promote stemness over tissue culture polystyrene plates.^89^ Increased cell–cell interaction markers platelet and endothelial cell adhesion molecule (*PECAM*) and integrin subunit alpha 2 (*ITGA2*) suggest 2D fibers promote cell–cell interactions over cell–matrix interactions and improved hMSC stemness compared to traditional culture plates.^89^ Maintaining stemness with nanofibers emulates the stem cell niche and improves hMSC function for therapeutic use.^89^

Evaluation of morphology includes measurements such as cells size and shape; cell, cytoskeleton, and nuclear alignment; or focal adhesion number and size. MSCs and tendon fibroblasts, among other cell types, demonstrate greater elongation,^84,86,90^ a greater aspect ratio,^84,85^ greater alignment,^84^ and a larger cell area^86,90^ on microfiber diameters (1–4 μm) compared to nanofibers (40–700 nm). However, there are exceptions where hMSCs^80^ and endothelial cells^91^ had larger surface areas on smaller fibers and cases where the fiber diameter did not affect rat MSC (rMSC) area^85^ or human U-87 glioblastoma multiform cell elongation or aspect ratio.^92^ hASCs,^79^ hMSCs,^80,81^ and human dermal^93^ and tendon^86^ fibroblasts have larger focal adhesions – clusters of proteins linking the ECM to the actin cytoskeleton – on microfibers compared to nanofibers, while human umbilical vein endothelial cells (HUVECs) increase adhesion to nanofibers.^94^ Focal adhesions of human dermal fibroblasts were larger but fewer on microfibers compared to nanofibers.^93^ In contrast, the focal adhesion clusters of C2C12 myoblast cells were longer on smaller compared to larger fiber diameters.^95^ Microfibers increased F-actin expression and enhanced actin organization.^86,93^ Endothelial cells on nanofibers expressed greater vascular endothelial cadherin, a cell–cell junction linking the actin cytoskeletons of adjacent cells.^91^ In the nanofiber range, rNSC morphology resembled oligodendrocytes,^87^ while on microfibers, rNSC morphology resembled neurons and neural progenitors;^87^ microfibers also promoted neurite extension and alignment.^96^ Generally, larger fiber diameters led to larger focal adhesions, which promotes actin organization and greater cell elongation, alignment, aspect ratios, and larger surface area.

While there are multiple types of cell migration, migration by protrusion of the actin cytoskeleton was the most common type identified in this review. In protrusion-based migration, the ‘finger-like’ actin bundles known as filopodia extend the membrane and sense the environment surrounding the cell. The ‘sheet-like’ actin network (the lamellipodia) then move the cell’s leading edge forward behind the filopodia as myosin contracts, sliding the trailing edge forward. Cells migrate at higher velocity on nanofibers (200–700 nm) than microfibers (1.1–5.7 μm).^92,95,96^ However, sometimes, the relationship reversed at higher structural stiffness, and C2C12 cells migrate faster on microfibers (1.2 μm) than nanofibers (400–700 nm).^95^

Cell migration is not restricted to a single fiber when the opportunity exists to interact with other fibers. At the subcellular level in NIH 3T3 fibroblasts, filopodia on larger nanofibers (~750 nm) sampled other fibers continually, but the lamellipodia extended along one fiber. On smaller nanofibers (~150 nm), the lamellipodia no longer continued along a single fiber when filopodia encountered other fibers.^90^ ND7/23 neuronal cells increased directionality on microfibers (5 μm) compared to larger nanofibers (~740 nm), on which the cells extended more radially outward.^96^ Embryonic dorsal root ganglia cells traveled greater distances, up to 1.5 mm on larger fibers than on smaller fibers.^51^ Similarly, osteoblastic MG63 cells penetrated further into scaffolds with larger fibers (4 μm) than smaller fibers (600 nm).^97^ However, the average pore size of larger fibers was also 10-fold greater than the smaller fibers, which suggests porosity mediates this effect and not fiber diameter.^97^ Together these data suggest that cells attain a higher peak velocity on smaller fibers but travel greater distances on larger fibers.

## General fiber parameters: Fiber alignment

Fiber alignment provides an inductive environment for certain lineages. For example, tenogenic markers, such as tenomodulin,^44,45,98,99^ scleraxis,^45,99,100^ tenascin-C,^99^ mohawk^45^ and type I collagen^99^ are increased in hASCs,^98,99^ tendon fibroblasts,^45^ and human tendon stem/progenitor^100^ cells on aligned fibers compared to randomly oriented fibers. Interestingly, hMSCs on aligned nanofibers without differentiation media required tensile, mechanical stimulation – besides fiber alignment – to induce a tenogenic/fibroblastic differentiation.^101^ hMSCs increased expression of markers for the superficial zone of cartilage on aligned fibers.^102^ hMSCs increased expression of osteogenic markers *RUNX2*, *ALP*, and *OCN* on aligned parallel electrospun fibers more than on orthogonally aligned fibers,^46^ possibly due to increased intercellular communication via gap junctions. In contrast, human skeletal stem cells increased mineral production and ALP activity on MEW fibers patterned at 90° angles more than those angled at 45° or 10°, or randomly oriented fibers.^58^ Furthermore, hASCs stained for more OCN on aligned graphene 2D fiber systems than on grid patterns, which the authors suggested was caused by increased cell elongation.^103^ While there was no significant difference between myogenic differentiation of C2C12 cells on aligned or randomly oriented microfibers,^104^ C2C12 myogenesis increased on aligned nanofibers compared to randomly oriented fibers.^105^ Aligned fibers guide hMSCs toward cardiomyogenesis^106^ and improve myoblast differentiation compared to randomly oriented fibers.^50^ Aligned fibers promote greater neural differentiation compared to random fibers.^52,107,108^ Aligned fibers promote the maturation of neuronal cells,^107^ but randomly oriented fibers provide a better environment for differentiation into glial cells.^108^

Fiber alignment has no effect on cell proliferation in human ligament fibroblasts,^109^ HUVECs,^110^ human aortic smooth muscle cells,^111^ human induced pluripotent stem cells,^112^ or U373 astrocytoma cells.^113^ However, human rotator cuff fibroblasts increase proliferation on non-aligned nanofibers.^86^ Similarly, corneal epithelial cells proliferated more on non-aligned fibers, while keratocytes proliferated more on aligned fibers.^114^ hMSCs proliferate more on aligned fibers than on randomly oriented fibers.^101,115^ Together, fiber alignment can enhance cell proliferation but is cell-type dependent.

Cells typically form an elongated, spindle-shaped morphology on aligned fibers,^85,91,92,113^ with alignment along the fiber direction^91,92,101,113^ as cytoplasm and F-actin colocalize along the fibers’ free boundary, maximizing contact.^91^ HUVECs increase alignment on aligned microfibers compared to aligned *nano*fibers due to increased contact guidance.^94^ Cells on aligned fibers increased vinculin^91^ and paxillin^86^ expression, indicating enhanced adhesion on aligned fibers. Neurite formation increased, with greater neurite extension^108,116^ on aligned fibers. In contrast, on randomly oriented fibers, cells typically form a round^85,92,113,117^ or polygonal^91,113,115,117^ morphology, with the cells exhibiting random orientation.^85,100,118^ The small pores between randomly oriented fibers reduce cell contact area and prevent cell elongation.^92^ Cells reorganized their actin cytoskeleton along aligned fibers’ boundary, resulting in an elongated cell shape oriented with the underlying fibers. However, on randomly oriented fibers, cells spread in all directions, resulting in a rounded morphology with random orientation.

Fiber alignment drives cell migration more than chemotactic gradients.^119^ Various cell types (hESCs,^52^ U87 MG glioblastoma multiform,^92^ and hMSCs^117^) migrate in the direction of fiber alignment. hMSCs,^117^ hESCs,^52^ NIH 3T3 fibroblasts,^120^ tumor-associated fibroblasts,^121^ HT-1080 human fibrosarcoma cells,^122^ murine MSC C3H/10T1/2 cells,^122^ and MDA-MB-231^123^ cells travel faster and farther on aligned fibers than non-aligned fibers. Further, cells migrate in the direction of aligned fibers, regardless of fiber diameter.^92^ Cell speed directly correlates with fiber alignment.^123^ Glioma cells migrated more on aligned fibers, while migration remained low on randomly oriented fibers except at low fiber density.^124^ Adult human dermal fibroblast migration velocity on thin films was initially high but decreased over 24 h and then remained constant. Whereas, the migration velocity of dermal fibroblasts on aligned fibers was low initially, but doubled over 4 days.^125^ Therefore, aligned fibers guide cell migration directionality and increase velocity and distance.

## General fiber parameters: Scaffold porosity

Mouse cardiosphere-derived cells increase cardiomyogenesis on high fiber density scaffolds.^126^ Conversely, NSCs increase proliferation and glial cell differentiation on high porosity/low fiber density scaffolds.^108^ Indeed, pore size (ranging from 900 nm to 40 μm) had a greater effect on cell proliferation than fiber diameter (ranging from 700 nm to 8 μm).^127^ Further, human dermal fibroblasts proliferated more on small pores (6 μm) than large pores (20 μm).^127^ Macroporous alginate scaffolds (pore size ~120 μm) enhanced MSC paracrine secretion by promoting cell–cell interactions (increased N-cadherin) compared to nanoporous scaffolds (pore size ~5 μm).^128^ Pore shape influences osteochondral differentiation. In a 3D-printed, scaffold with constant fiber diameter, square pores supported greater chondrogenic differentiation, while rhomboidal pores supported greater osteogenic differentiation, with osteochondral media.^129^ Thus, both pore size and shape drive cell differentiation and proliferation. The effect of fiber density and scaffold porosity on tenogenic, adipogenic differentiation and a range of other differentiation pathways are not well understood.

On pore sizes of similar dimensions to cell size, HEK293T cells exhibited minor pseudopodia but had obvious pseudopodia extension on pores larger than cell size.^130^ With aligned fibers spaced on similar scales of cell-size (10 μm or 20 μm), HEK293T cells demonstrated aligned, nanofiber-dependent orientation behavior: cells guided along fibers, resulting in nanofiber-dependent bipolar cell morphology.^130^ With larger spacing (40 μm or 80 μm), cells clustered but aligned with fibers at the cluster edges, while in the middle of the cluster, cells were randomly aligned.^130^ HUVECs spread more on fiber scaffolds with greater fiber density, i.e., lower porosity.^110^ On larger pore sizes, human dermal fibroblasts attached to individual fibers, rather than spread across multiple,^127^ while hMSCs aligned more on high-density fibers.^46^

Larger pore size increased migration speed of HT-1080 human fibrosarcoma on 3D fibers^123^ and also increased osteoblast motility on aligned 2D fiber systems.^131^ Similarly, cell migration distance increased on low density/high porosity electrospun fibers for human lymphatic endothelial cells.^132^ Large pores and high porosity drive increased migration speed and distance. As cells attached to single fibers in scaffolds with large pores,^127^ the large pores leave the cells with one option – follow the only fiber available – leading to increased migration speeds. Smaller pores (6.5±3.3 μm) prevented fibroblasts from migrating into the scaffolds.^133^ Additionally, cells on randomly oriented fibers with small pores adhered to multiple fibers – across the pores – which prevented them from forming an elongated, polarized shape found in migrating cells.^92^ Therefore, larger pores force the cell alignment along a single fiber, giving cells few options on where to migrate.

## General fiber parameters: Fiber mechanical properties

In addition to the fiber parameters, the mechanical properties of the fibers can guide cell response. Properties such as stiffness and Young’s modulus – affect cell differentiation,^134^ morphology,^135^ and migration.^136^ While the mechanical properties of the individual fibers are inherent to the material used, the mechanical properties of fibrous scaffolds depend on both the materials’ mechanical properties as well as the fiber architecture. Fiber diameter is inversely correlated with Young’s modulus.^75^ Fiber alignment has a ‘U-shaped’ effect on Young’s modulus across the nano-macroscale, with a global maximum at perfectly aligned fibers and local maxima when the fibers are orthogonal.^137^ Fiber porosity is also inversely correlated to Young’s modulus.^138^ Therefore, the mechanical properties of fibrous scaffolds should be accounted for during scaffold design.

hMSCs exhibited greater expression of chondrogenic markers after 28 days in culture on soft (poly(ε-caprolactone) (PCL); 25–30 MPa) and medium (poly(lactic acid) (PLA); 80–90 MPa) stiffness materials than on stiff (poly(glycolide) (PGA); 150–160 MPa) materials with the same structure and architecture,^83^ including increased expression of type II collagen; middle/deep zone cartilage markers cartilage intermediate layer protein, cartilage oligomatrix protein, and type IX collagen; and superficial zone cartilage marker proteoglycan 4. Type I collagen expression was upregulated on the stiffest (PGA) substrate.^83^ The PGA substrate degraded more than the PLA or PCL scaffolds (23% vs 15% and 10%, respectively) but altered only ~5% of the surface architecture. While the PGA degradation did not affect hMSC response via decreased scaffold alignment, no further effects of degradation on cell response were investigated.^83^ Direct insertions of tendon into bone, known as the enthesis, have a gradient of structural and mechanical properties across a tendinous zone, a fibrocartilage zone, a mineralized fibrocartilage zone, and bone. rASCs cultured on a poly(lactic-co-glycolic acid) (PLGA) scaffold designed to reflect the enthesis – with distinct zones that increase in stiffness (200 MPa to 2 GPa) and mineralization (hydroxyapatite) while transitioning from aligned fibers mimicking the collagen in tendon (50 μm diameter; 150 μm in spacing) to an interconnected network of pores mimicking the structure of cartilage and bone (200 μm pore diameter) – differentiated into tendon-like cells in the less stiff, unmineralized regions whereas the rASCs differentiated toward osteogenic lineages in the higher stiffness, mineralized regions of the scaffold.^139^ rMSCs differentiated more toward ligament-like lineages on electrospun nanofibers with lower stiffness (5.6 MPa vs 15 or 31 MPa) but similar fiber diameters (0.66–0.77 μm) and alignment (13–21° angular standard deviation).^140^ NSCs differentiated into neuronal cells on fibers (1.5–2 μm diameter) with a higher elastic modulus (99 MPa), high fiber alignment, and low porosity (46%) but differentiated into glial cells more on fiber systems with a lower elastic modulus (35 MPa), lower alignment, and higher porosity (86%).^108^ As alignment and porosity were not controlled for, it is difficult to determine to what extent the mechanical properties of the fibers guided NSCs toward different lineages. Smooth muscle cells (SMCs) were observed to exhibit greater proliferation on a 3:1 poly(urethane):collagen blend that had a greater elastic modulus (27.5 MPa) than other fibers with similar fiber diameters, alignment, porosity and water contact angles but lower elastic moduli (4–20 MPa).^111^

Cells spread more on fibers with greater stiffness (stiffness range of 1.8 MPa to 1.1 GPa),^141,142^ with greater cell adhesion,^142^ and higher aspect ratios.^141^ On aligned nanofibers (~700 nm fiber diameter), MSCs oriented with the fibers more on less stiff fibers (5.6 MPa) compared to stiffer fibers (15 MPa and 31 MPa).^140^ Substrate stiffness, and not topography, modulated nuclear area within Chinese hamster ovary cells, while the nuclear shape was modulated by substrate topography, and not stiffness.^142^ C2C12 cells and their nuclei elongated while decreasing their width as stiffness increased from 2mN/m to 120mN/m, while the length of paxillin focal-adhesion-clusters increased as stiffness increased.^95^

On single poly(dimethylsiloxane) (PDMS) Spinneret-based Tunable Engineered Parameters (STEP) fibers, C2C12 cells exhibited migration speeds inversely correlated to stiffness – regardless of fiber diameter (tested at 400 nm, 700 nm, 1200 nm).^95^ The stiffness of the PDMS STEP fibers increased along the length of the fiber (20 mN/m to 60 mN/m for the 400 nm-diameter fibers; 5 mN/m to 25 mN/m for the 700 nm-diameter fibers; 1 mN/m to 15 mN/m for the 1200 nm-diameter fibers), and the majority of C2C12 cells migrated toward the regions of higher stiffness.^95^ However, on collagen fibers, HT-1080 human fibrosarcoma cell migration speed and invasiveness did not correlate with matrix stiffness (elastic modulus ranging from 100 Pa to 500 Pa) over the range of collagen concentrations tested.^123^

## General fiber parameters: Surface chemistry

High surface roughness (roughness average (Ra) = 71±11 nm) increased the expression of osteoprogenitor markers bone morphogenetic protein 2 (*BMP2*) and runt related transcription factor 2 (*RUNX2*), as well as osteoblast marker osteopontin, in hMSCs, while a low surface roughness (Ra = 14.3±2.5 nm) increased the expression of osteoblast markers osteopontin, type I collagen, and osteocalcin.^143^ Scaffolds exhibiting medium levels of roughness (Ra ~40 nm) increased the expression of chondrogenic markers *SOX9*, collagen type II (*COL2A1*), and *ACAN* in hMSCs compared to lower or higher surface roughness (~14 nm and ~70 nm, respectively).^143^

Surface wettability (hydrophobicity and hydrophilicity) affects differentiation and proliferation. Octadiene-allylamine polymers modified with a gradient of increasing allylamine (to increase hydrophilicity) increased mouse ESC proliferation and differentiation toward mesodermal and ectodermal lineages, while the same polymer surface modified with methyl groups to increase hydrophobicity did not promote mouse ESC differentiation or proliferation. Surface wettability did not have an evident effect on mouse ESC differentiation toward endodermal lineages.^144^ Hydrophilic surfaces promoted human dental pulp stem cells (HDPSCs) toward osteogenic lineages, increasing *RUNX2*, *ALP*, dentin sialophosphoprotein, and dentin matrix acidic phosphoprotein 1 gene expression, as well as ALP activity.^145^ Osteoblasts proliferated more on hydrophilic surfaces, while also increasing ALP activity and osteocalcin production.^146^ C2C12 myoblasts increased myogenic protein expression on hydrophilic surfaces compared to hydrophobic surfaces.^147^ However, on super-hydrophilic surfaces (water contact angle <5%), myogenic expression decreased in C2C12 cells.^147^

Surface roughness affects cell morphology. ADTC5 chondrogenic cells and SaOs-2 (‘sarcoma osteogenic’) cells had a more spread out, flatter morphology on smooth surfaces (Ra = 13 μm), but had a more rounded morphology on rough surfaces (Ra = 21 μm).^148^ Surface wettability can also affect cell morphology. Hydrophilic surfaces modified with amine groups led to increased branching and osteocyte-like morphology in HDPSCs, while HDPSCs maintained an MSC morphology on the more hydrophobic surfaces modified with methyl groups and hydrophilic surfaces modified with hydroxyl or carboxyl groups.^145^

Rougher surfaces promote cell migration over smoother surfaces.^149^ Poly(methyl methacrylate) surfaces were sandblasted to increase surface roughness. The increased roughness led to a two-fold increase in migration area in vascular cells, as well as a three-fold increase in migration area in corneal cells. Additionally, hydrophobic surfaces promote cell migration in vascular endothelial cells and corneal cells. Conversely, hydrophilic surfaces promote cell adhesion.^145,146^ Hydrophilic surfaces increased the expression of focal adhesion proteins talin and paxillin.^150^ Hydrophobic surfaces, however, increased expression of Rho GTPases ras homolog family member A (RhoA), rac family small GTPase 1 (Rac1), and rho-associated, coiled-coil containing protein kinase 1 (ROCK) (a downstream effector of RhoA), which facilitate migration.^150^

## Immune response to fiber diameter and alignment

Fiber diameter affects immune response, with lower classical M1 macrophage activation^151^ and lower proinflammatory cytokine secretion from macrophages^151,152^ and endothelial cells^153^ on smaller fiber diameters (200–600 nm) than larger fiber diameters (1–50 μm). Peak macrophage elongation occurred on smaller fibers.^152^ While the fiber diameter affects the immune response, fiber alignment is not as important.^151,153^ However, fiber alignment influences the response to inflammatory signals.^45^ When stimulated by macrophage paracrine signaling, tendon fibroblasts increased matrix production, as well as matrix metalloproteinase and tissue inhibitor of metalloproteinase expression, on randomly oriented fibers compared to tendon fibroblasts on aligned fibers.^45^ The influence of fiber microarchitecture on other immune cell populations is not well understood.

## Dynamic scaffolds

Recent studies have investigated cell behavior in response to dynamic scaffolds that mimic the ever-changing, *in vivo* environment.^122,154,155^ Dynamic scaffolds using shape-memory polymers change fiber diameter, alignment, and porosity of a scaffold reversibly via temperature^155^ change or magnetic fields.^154^ As environments changed from randomly oriented fibers (or channels) to aligned fibers, cells aligned with the fibers; the cell morphology changed from a rounder shape on the random fibers to an elongated spindle shape in the aligned environment.^122^ The area and shape of A7R5 rat smooth muscle cells changed with acute changes in topography roughness, but morphology remained constant over extended periods of oscillation.^154^ Dynamic scaffolds also offer the advantage of changing shape after implantation,^155^ allowing for small, easily implantable scaffolds that can grow to fill a tissue defect while preserving the ability to guide cells toward the desired lineage.^155^ As shape memory polymer technology continues to advance, it offers a promising means to mimic the dynamic ECM further and regulate cell response in other cell lines and differentiation pathways.

## Incorporating biomimetic factors into synthetic scaffolds

While fiber parameters can drive cell responses, a limitation of synthetic polymer scaffolds is the lack of cell signaling cues provided by the native ECM. Therefore, tissue engineering commonly incorporates proteins and other biomimetic factors from the ECM into synthetic polymer scaffolds to provide additional cell signaling cues to the enhanced structural and architectural cues provided by synthetic scaffolds. These biomimetic factors are incorporated through various means: coating a scaffold, covalently linking to the scaffold, or by adding nanoparticles to the system that release the biomimetic factors over time. In all cases, the addition of biomimetic factors seeks to further enhance and guide cell response to the desired end.

Incorporating growth factors and other biomimetic molecules into the fiber system is commonly used to further induce differentiation using various strategies. Coating poly(L-lactic acid) (PLLA) electrospun fibers with polydopamine induced osteogenic differentiation in hMSCs, with greater expression of *ALP*, *RUNX2*, bone sialoprotein, and interleukin 8 on the polydopamine-coated fibers than on the uncoated fibers – both scaffolds with comparable fiber parameters.^156^ Adding hydroxyapatite and graphene oxide to electrospun PLGA fibers increased ALP activity, RUNX2, and OPN expression, calcium deposition, and cell proliferation in mouse MC3T3-E1 pre-osteoblast cells, but also decreased the fiber diameter of the scaffolds from 1.35 mm to 885 nm.^157^ As smaller fiber diameters also promote osteogenic differentiation,^80,81^ this decrease in fiber diameter could equally have affected the increased osteogenic gene expression. The addition of graphene oxide to the fibers increased their tensile strength two-fold. The increased mechanical properties could be the mechanism leading to the increased osteogenic expression, as high substrate elasticity and stiffness guide cells toward osteogenic lineages.^134^

The effect of transforming growth factor beta 3 (TGF-β3) in culture medium depended on fiber alignment: on aligned fibers (4^ο^ angular deviation; 5.2 μm fiber diameter; 76% porosity), TGF-β3 induced chondrogenesis in hMSCs via increased *COL2A1*, *ACAN*, and *SOX9* expression, whereas TGF-β3 induced osteogenesis in hMSCs (increased *BMP2*, *RUNX2*, and *COL1A1* expression) on randomly aligned fibers (5.1 μm fiber diameter; 79% porosity).^44^ Aligned fibers coated with connective tissue growth factor increased tenomodulin expression inducing ligamentous or tenogenic differentiation of hMSCs.^44^ Incorporation of nanoparticles (~150 nm) containing platelet-derived growth factors (PDGF) into aligned collagen fibers increased tenomodulin and scleraxis expression in rASCs, with no effect on unwanted ALP activity or osteocalcin production.^98^ However, the PDGF-nanoparticles did not increase tenogenic expression in rASCs cultured on randomly oriented fibers.^98^ While PDGF was found to improve tenogenesis on aligned collagen fibers but not randomly oriented fibers, the fiber diameters and porosity were not investigated or controlled.^98^

Bioprinting is yet another method to include growth factors to influence differentiation. Aligned nanofibers promote osteogenesis,^46,80,81^ tenogenesis,^45,98,99^ and myogenesis^158^ depending on cell type. Bioprinting growth factors onto polystyrene STEP fibers (~668 nm fiber diameter; angular deviation of 2.5^ο^) modulated cell fate: C2C12 cells differentiated toward tenogenic lineages on regions coated with fibroblast growth factor 2, toward osteogenic lineages in regions coated with bone morphogenic protein (BMP)-2, but toward myogenic lineages in uncoated regions.^158^

Incorporation of proteins into the fiber system also enhances differentiation. Human aortic SMCs did not proliferate on randomly oriented or aligned polyurethane nanofibers, but the addition of collagen into the fibers increased SMC proliferation.^111^ However, while the fibers were nanofibers in both cases, the polyurethane:collagen-blend fibers had a decreased fiber diameter (~200 nm vs. ~400 nm) and decreased pore size (~300 nm vs. ~700 nm), which could have promoted proliferation but it was not controlled for.^111^ Blended PLLA/type I collagen (4:1 ratio) nanofibers led to increased type I, II, and X collagen, and decorin expression at 4 days.^159^ However, the PLLA/type I collagen fiber diameters (238 nm) were lower than the PLLA-alone fibers (750 nm), which could affect the gene expression, but was not investigated.^159^

Many biological tissues exhibit endogenous electric fields, which have been characterized during development and regeneration.^160^ Cartilage^161^ and bone^162^ exhibit piezoelectric behavior during loading, but piezoelectric biomaterials remain relatively unexplored in tissue engineering.^163^ Recently, however, electrospun fibrous scaffolds composed of piezoelectric material were designed and investigated in the chondrogenesis and osteogenesis of hMSCs.^163^ Piezoelectric fibers (5.9 μm fiber diameter; 93% porosity) with a low voltage output produced an electric field that (20 mV/mm) promoted chondrogenesis while electrospun PCL (9.8 μm fiber diameter; 88% porosity) scaffolds could not. Alternatively, piezoelectric fibers (6.9 mm fiber diameter; 92% porosity) with a high voltage output produced electric fields that (1 V/mm) promoted osteogenesis compared to the low voltage piezoelectric fibers and the PCL fibers.^163^ The electric fields, along with mechanical stimulation, improved differentiation compared to mechanical loading alone.^163^

Coating aligned, electrospun nanofibers with fibronectin improved neurite extension in NG108-15 neuroblastoma and glioma cells compared to the uncoated fibers.^116^ hMSCs elongated on polydopamine-coated fibers – even on randomly oriented fibers where they normally form rounded morphologies.^117^ hMSCs demonstrated greater spreading,^117^ enhanced adhesion,^117,156^ and robust focal adhesion formation^156^ on polydopamine-coated fibers compared to uncoated fibers. Human skeletal muscle myoblasts and fibroblasts elongated on nanofibers coated with laminin or collagen while they exhibited polygonal morphology on the uncoated fibers.^164^ Porous electrospun fibers enhanced PC12 cell adhesion.^118^ L929 cells protruded more pseudopodia, and many filopodia anchored into the pores, suggesting porous fibers can enhance cell adhesion.^118^ NIH 3T3 fibroblasts aligned more with larger, aligned PLGA fibers (740 nm diameter) than with smaller, aligned fiber (140 nm diameter).^90^ On the larger fibers, the cells’ filopodia continuously sampled other fibers, but their lamellipodia extended primarily along single fibers. On the smaller fibers, however, the fibroblasts’ lamellipodia extended along many directions when their filopodia contacted misaligned fibers, likely due to the decreased adhesion sites on the smaller fibers. Indeed, when the PLGA fibers were treated with poly(L-lysine) to improve cell adhesion, the fibroblasts’ alignment significantly increased – especially on the smaller fibers – suggesting that more stable focal adhesion complexes guide cell alignment.^90^

Mouse E13 NSCs’ migration distance on fibers coated with poly-D-lysine depended on fiber diameter, while coating the fibers with laminin induced promoted migration regardless of fiber size.^165^ NSC neurospheres migrated radially outward equidistantly on laminin-coated nanofibers (860 nm) but extended along the direction of the fibers on laminin-coated microfibers (8.8 μm).^165^ In 3D culture, HT-1080 fibrosarcoma cells traveled long distances, rapidly and persistently, while maintaining high protrusion formation rates on low collagen densities that had increased alignment and pore sizes (1 mg/mL).^123^ Cell migration slowed on intermediate collagen densities (decreased fiber alignment and pore size compared to the low-density collagen fibers) but then increased on higher collagen densities (6 mg/mL) despite no significant changes in fiber alignment or pore size compared to the intermediate collagen densities.^123^ The opposite response occurred for 2D cell motility with increasing ligand density.^123^ This same biphasic pattern arose in MDA-MB-231 cells.^123^ Crosslinking the collagen reduced cell migration speed without altering the collagen gel density,^123^ adding further evidence that fiber alignment is a significant factor in cell migration, even with biomimetic factors. Topographical cues from fiber alignment dominated HUVEC motility over a chemical gradient of vascular endothelial growth factor orthogonal to fiber alignment but had an additive effect when the two were parallel.^119^ Fiber diameters and porosity were not reported in the study, but the fibrous scaffolds were electrospun using the same parameters and should have similar architecture.^119^

## Conclusions

The literature demonstrates that fibers drive many cellular responses. In native ECM, the fibrous proteins provide signaling cues to drive cell differentiation, proliferation, adhesion, and migration. Tissue engineering controls the fiber parameters of scaffolds to regulate cell response during engineered development (Table 3). Fiber diameter regulates differentiation in a lineage-dependent manner: nanofibers drive osteogenesis, fiber diameter has a biphasic effect on chondrogenesis, the effect of fiber diameter on tenogenesis changes over time, and differing fiber diameters can drive cells toward specific neural lineages. Larger fiber diameters lead to greater cell elongation and alignment. Cells migrate at higher speeds on smaller fibers, while they migrate farther distances on larger fibers. Increased fiber alignment can drive cells into tenogenic, cardiomyogenic, and neuronal lineages, while non-aligned fibers guide cells toward osteogenic and glial differentiation. Cells elongate and align with underlying fibers, forming a spindle shape morphology on aligned fibers, while they form a rounded morphology on randomly oriented fibers. Therefore, aligned fibers appear to provide a cellular ‘highway’: cells migrate along the direction of aligned fibers and migrate faster on aligned fibers. Cells will follow aligned fibers preferentially across chemotactic gradients. Low scaffold porosity, or high fiber density, leads to greater cell proliferation. Porosity also guides differentiation into multiple cell lineages as a function of pore size and shape. When the pores are small, cells can extend across multiple fibers, leading to a more rounded morphology and lower migration speed. Conversely, when the pores are large, cells will attach and align with single fibers, which also results in increased migration speeds. While synthetic fibers can drive these responses, incorporating biomimetic factors into the scaffolds can further improve the desired response and modulate the response via interactions with the scaffold structure and architecture.

**Table 3:** General trends of cell response to fiber parameters

Fiber Parameters	Differentiation	Morphology	Migration
Diameter			
Nanofibers	Osteogenesis Chondrogenesis Tenogenesis Myogenesis Neurogenesis (Glial)	Rounded Larger focal adhesions	↑ Velocity
Microfibers	Adipogenesis Chondrogenesis Tenogenesis Neurogenesis (Neuronal)	↑ Elongation ↑ Aspect Ratio ↑ Alignment ↑ Area More focal adhesions	↑ Distance
Alignment			
Random	Neurogenesis (Glial)	Round Polygonal Random Orientation	
Aligned	Osteogenesis Tenogenesis Myogenesis Neurogenesis (Neuronal)	↑ Elongation ↑ Alignment Spindle Shape Cytoskeletal Alignment	↑ Velocity ↑ Distance Direction of Fibers
Porosity			
Low	Myogenesis	Rounded ↑ Spreading Attach to Multiple Fibers	
High	Neurogenesis (Glial)	↑ Elongation Larger Pseudopodia Attach to Single Fibers	↑ Velocity ↑ Distance

Cells tend to align and elongate along microfibers and aligned fibers through common intracellular mechanisms. Commonly on both microfibers and aligned fibers, cells formed larger or greater numbers of focal adhesions along the increased cell-contact area. This also occurred in cells along large pores (i.e., essentially along a ‘single fiber’ in all cases). The increased focal adhesions lead to the actin cytoskeleton aligning along the fibers, which generated the elongated, spindle shaped morphology. Similarly, cells migrated faster and further on aligned fiber ‘highways’ and fibers with large pores. In these cases, the cells’ lamellipodia sample along the ‘single fiber’ with little distraction from other directions, resulting in increased migration speeds. However, cells on microfibers had lower migration speeds than on nanofibers. Microfibers increase cell migration directionality (along the fibers) but saw lower cell velocities than nanofibers. Cells form larger focal adhesions on the microfibers than on the nanofibers, which generally predicts higher migration speeds.^166^ The intracellular mechanisms resulting in reduced cellular velocity on microfibers despite larger focal adhesions and increased directionality remains unknown.

The major limitation facing many current studies is the failure to consider all fiber parameters in toto, instead focusing only on one. Changing scaffold production to affect one parameter often changes others simultaneously, and if these other parameters are not adequately controlled, it raises questions about the effects seen. Many studies characterize fiber parameters and then look for a desired outcome, however, the mechanisms driving the outcomes are not investigated as often. Similarly, including biomimetic factors into fibrous scaffolds can change the structure and architecture of fibers and needs to be controlled. Additionally, material properties such as stiffness or wettability cannot be ignored when investigating the effects of fiber parameters on cell response.

## Future directions

While there have been substantial advances in our understanding of cell–fiber interactions, some remaining gaps include: (1) investigating the effect of a larger range of fiber diameters: many studies investigate the effects of nanofibers for cell differentiation and migration. However, there is growing evidence that microfibers guide cells toward specific lineages. Screening a wider range of fiber diameters from nanofibers to microfibers for a wide range of cell types could inform tissue engineering design to achieve the most desired outcome for a variety of tissues. (2) Determining the mechanisms that translate the physical cues from the fiber parameters into the biological signals that drive differentiation, morphology, and migration. (3) Determining the interactions between fiber parameters, mechanical properties, and surface chemistry: while expanding knowledge of fiber diameter’s effect on cells would benefit the field, it would require an assessment of how the other fiber parameters (alignment and porosity), along with material properties (mechanical and chemical) can optimize cell differentiation, morphology, and migration. (4) Incorporating biomimetic factors: as the scaffold’s physical parameters are optimized, the field needs to continue to develop and improve methods for incorporating biomimetic factors to induce relevant cell signaling. While doing this, the fiber parameters and material properties need to be considered, as they modulate cellular response to the biomimetic cues. (5) Designing heterogeneous scaffolds to grow tissues: combining the optimized scaffold architecture and biomimetic cues into scaffolds with heterogeneous regions could be used to culture ‘synthetic’ tissues to replace natural ones: tendons with a midsubstance region of aligned fibers populated by tenocytes transitioning into the fibrocartilaginous region of the enthesis populated by fibrochondrocytes that becomes mineralized as it transitions into bone; cartilage scaffolds with three distinct zones mimicking the varying structures superficial, middle, and deep zones; scaffolds that guide cells to form cortical and cancellous bone. While much work is still needed to achieve this feat of tissue engineering, understanding how fiber parameters guide cell response helps to pave the way.

### Reporting summary

Further information on research design is available in the Nature Research Reporting Summary linked to this article.

## Supplementary information

Reporting Summary Checklist

## Supplementary information

**Supplementary information** accompanies the paper on the *npj Regenerative Medicine* website (10.1038/s41536-019-0076-5).
